# Fueling the Future: Advances in Sports Nutrition for Young Athletes

**DOI:** 10.3390/nu18142327

**Published:** 2026-07-16

**Authors:** Filipa Vicente

**Affiliations:** Applied Nutrition Research Group (GENA), Nutrition Lab, CIIEM—Egas Moniz School of Health & Science, Quinta da Granja—Campus Universitario, 2829-511 Caparica, Almada, Portugal; fvicente@egasmoniz.edu.pt

Young athletes should not be considered smaller versions of adult athletes. Their nutritional needs are shaped by the simultaneous demands of growth, maturation, training, competition and recovery [1,2,3], often within environments where food choices are constrained by school timetables, family routines, travel, club resources and increasing exposure to performance-related pressures. This makes youth sports nutrition a distinct area of practice and research, requiring recommendations that are physiologically sound, developmentally appropriate and feasible in real-world sporting contexts.

This Special Issue, “Fueling the Future: Advances in Sports Nutrition for Young Athletes”, brings together contributions that address some of the key challenges in this field, including energy and nutrient requirements, nutrition education, supplement use and dietary strategies relevant to young or developing athletic populations. Collectively, these articles show that supporting young athletes requires more than optimizing short-term performance. It requires protecting growth and maturation, promoting food literacy, reducing unsafe practices and translating evidence into practical routines that athletes, families, coaches and clubs can realistically implement.

A central theme emerging from this Special Issue is nutritional adequacy during adolescence. Everett highlights that adolescent athletes face the simultaneous demands of growth, development and athletic performance, making energy and nutrient sufficiency essential for both health and performance [4]. Particular attention is given to energy availability, carbohydrate and protein intake, hydration strategies and micronutrients such as iron, calcium and vitamin D [2,3,5]. These issues are not merely theoretical. In practice, young athletes frequently fail to meet their nutritional requirements, not only because of intentional restriction, but also because of poor planning, limited nutrition knowledge, long school days, travel demands, appetite fluctuations after training and a lack of structured support within clubs.

Low energy availability deserves particular attention in youth sport. During adolescence, inadequate energy intake may compromise growth, pubertal development, menstrual function, bone health, immune function, recovery and training adaptation [6].

Carbohydrate availability remains another critical issue. Although adult sports nutrition increasingly discusses carbohydrate periodisation and metabolic flexibility [7,8], such strategies must be critically avoided in youth. The systematic review and meta-analysis by Gawelczyk et al. [9] contributes to the broader discussion on low-carbohydrate and ketogenic diets in trained athletes, but its implications for younger populations require caution. In youth athletes, carbohydrate intake must be considered in relation to training demands, recovery, school performance, growth and total energy availability. From an applied perspective, restrictive dietary strategies may be inappropriate when basic fuelling habits are not yet established, or when athletes are vulnerable to under-fuelling, body image concerns or externally imposed weight expectations.

Nutrition education is another important dimension of this Special Issue. In one of the contributions, our research group [10] evaluated a pilot nutrition education programme in Portuguese male youth football players and reported significant improvements in general and sports nutrition knowledge after an intervention combining face-to-face sessions and digital infographics. However, the same study found no significant improvement in adherence to the Mediterranean diet over the short intervention period. This distinction is important. Improving knowledge is necessary, but it should not be confused with achieving sustained behavioural change.

In applied settings, young athletes’ eating behaviours are shaped by several influences beyond individual knowledge. Parents often determine food availability and meal organisation; coaches may influence beliefs about body weight, recovery and supplements; peers and social media shape norms and expectations; and school and club routines frequently determine when and what athletes are able to eat. For this reason, nutrition education programmes should not be limited to the transmission of information. They should include practical food skills, meal planning, label interpretation, critical evaluation of supplement claims and strategies that involve the athlete’s wider support network [11].

The gap between knowledge and behaviour is particularly evident in food labelling and supplement use. Even when adolescents receive nutrition education, interpreting serving sizes, ingredients, claims and marketing messages can remain difficult. This is especially relevant because young athletes are increasingly exposed to commercial content that presents supplements as essential for performance. Jojić et al. [12] addressed supplement use in adolescents aged 14–18 years, highlighting the importance of education in a digital environment where young athletes may be influenced by social media content, peer norms and performance-related marketing. In this population, supplementation should therefore be framed primarily as an issue of literacy, safety and professional supervision, rather than as a shortcut to improved performance.

From a practical sports nutrition perspective, a food-first approach remains the safest and most appropriate foundation for young athletes. This does not mean rejecting all supplements, but it does require a clear hierarchy. Adequate energy intake, dietary quality, hydration, meal timing, recovery and sleep should be addressed before considering ergogenic aids. When supplementation is considered, it should be based on a clear rationale, evidence of need, age-appropriate safety considerations, product quality and professional oversight. This is particularly important during adolescence, when the pursuit of marginal gains can distract from the basic behaviours that matter most for health, development and performance.

The contribution by Dou et al. [13], which examined the effects of four weeks of Rhodiola rosea supplementation on intermittent exercise performance and decision-making under fatigue in football players, illustrates the growing interest in nutritional and bioactive strategies that may support performance maintenance, particularly under fatigue. The study reported improvements in Yo-Yo intermittent recovery performance, repeated-sprint mean time, post-exercise lactate responses and decision-making under fatigue. Nevertheless, such findings should be interpreted within the specific population, design and context of the study. For young athletes, ergogenic strategies must always be weighed against maturity, safety, regulation, product quality and the priority of establishing robust dietary foundations [14].

Adherece and implemmentation remains one of the main challenges in youth sport. Evidence generated under controlled conditions is valuable, but its translation into everyday sporting environments is often challenging. Young athletes may train early in the morning or late in the evening, travel long distances to competition, rely on parents for food provision, eat within school constraints or have limited access to appropriate recovery meals. Recommendations that are physiologically sound but practically unrealistic are unlikely to produce meaningful change. Sports nutrition professionals must therefore act not only as experts in nutrient metabolism, but also as translators of evidence into simple routines, feasible food choices and sustainable behaviours.

The surrounding environment is decisive. Coaches, parents and clubs can either facilitate or undermine appropriate nutritional practices. Coaches may influence beliefs about body weight, leanness, supplement use and acceptable eating behaviours [15]. Parents often control food purchasing, meal preparation and transport logistics [16]. Clubs shape food availability, recovery routines and the implicit culture around fuelling. Therefore, the nutritional care of young athletes should be interdisciplinary and ecological. Effective intervention requires not only educating the athlete, but also aligning the messages delivered by families, coaches, health professionals and sporting organisations [17].

Despite the growing body of research, important gaps remain. Studies in youth sports nutrition are frequently limited by small samples, heterogeneous sporting populations, cross-sectional designs, short intervention periods and variable methods of dietary assessment. Future research should prioritise longitudinal designs, pragmatic interventions in real-world sporting environments and outcomes that go beyond short-term performance metrics.

A final challenge is the need to balance performance ambitions with safeguarding. Youth sport can provide a powerful setting for developing discipline, physical literacy, confidence and lifelong health behaviours. However, it can also expose young athletes to inappropriate dietary restriction, supplement misuse, body dissatisfaction and harmful weight-control practices. Nutrition support in youth sport must therefore include a clear safeguarding dimension. The objective is not merely to optimise the next training session or competition, but to support the young athlete’s long-term development as both an athlete and a healthy individual.

In conclusion, this Special Issue highlights the need to recognise youth sports nutrition as a distinct field, rather than an extension of adult sports nutrition. Supporting young athletes requires strategies that integrate performance with growth, maturation, health, recovery, education and safety. Further research is urgently needed to build robust, context-sensitive evidence capable of guiding practice in real-world youth sport settings. Advancing this area is essential to protect young athletes’ health while supporting their long-term development and athletic potential.
